# Simvastatin Induces Apoptosis but Attenuates Migration in SCAPs

**DOI:** 10.1016/j.identj.2023.10.015

**Published:** 2024-01-14

**Authors:** Paak Rewthamrongsris, Suphalak Phothichailert, Uraiwan Chokechanachaisakul, Chatvadee Kornsuthisopon, Thanaphum Osathanon

**Affiliations:** a Center of Excellence for Dental Stem Cell Biology and Department of Anatomy, Faculty of Dentistry, Chulalongkorn University, Bangkok, Thailand; b Department of Operative Dentistry, Faculty of Dentistry, Chulalongkorn University, Bangkok, Thailand

**Keywords:** Stem cells from apical papilla, Simvastatin, Cell viability, Cell proliferation, Cell migration

## Abstract

**Aim:**

Simvastatin has emerged as having a promising role in controlling stem cell behaviours. This study aimed to evaluate the effects of simvastatin on the viability, growth, and migration of stem cells isolated from apical papillae (SCAPs) *in vitro*.

**Methods:**

SCAPs were isolated and characterised. The viability and proliferation were assessed using live/dead and 3-(4,5-dimethylthiazol-2-yl)-2,5-diphenyl-2H-tetrazolium bromide (MTT) assays, respectively. Cell migration was evaluated using scratch assays. Cell cycle progression and apoptosis were examined using flow cytometry analysis.

**Results:**

Simvastatin at a concentration of 100 to 1000 nM did not exhibit cytotoxicity. Simvastatin reduced cell numbers at days 3 and 7. In addition, simvastatin markedly decreased colony formation in both colony number and cell density in a dose-dependent manner. An increase in apoptosis was observed at day 7. There was statistically significant increased in sub G0 population. An *in vitro* cell migration was attenuated in a dose-dependent manner.

**Conclusion:**

Simvastatin affects SCAPs’ viability, proliferation, and cell migration. The reduction of cell viability at day 7 could be due to apoptotic induction.

## Introduction

An inhibitor of 3-hydroxy-3-methylglutaryl coenzyme A reductase or statin is a medication used for lowering blood lipid levels. Simvastatin has emerged as a promising candidate to modulate stem cell behaviour. Numerous studies have provided evidence that simvastatin can affect cell proliferation and promote osteogenic differentiation in various types of stem cells, including dental pulp stem cells (DPSCs) and bone marrow–derived mesenchymal stem cells.^1^, ^2^, ^3^, ^4^, ^5^ Simvastatin can induce odonto-/osteogenic differentiation of DPSCs both *in vitro* and *in vivo*. In this regard, simvastatin suppressed DPSC proliferation but markedly increased mineral deposition as well as alkaline phosphatase enzymatic activity in vitro. Simvastatin significantly upregulates the dentin sialophosphoprotein (*DSPP*) gene, but not bone morphogenetic protein-2 (*BMP2*) expression.^4^ Simvastatin induces regeneration of the coronal pulp in pulpotomised teeth.^5^ The combination of stem cells isolated from human exfoliated deciduous teeth (SHEDs) with simvastatin-loaded microspheres promotes endothelial cell migration and angiogenesis in vitro. Furthermore, subcutaneous implantation illustrated that this combination regenerates vascular-rich dental pulplike tissue.^6^

In regenerative endodontic treatment, stem cells isolated from apical papillae (SCAPs) are a significant population known for their ability to withstand pulpal inflammation and necrosis, contributing to the regeneration of dentin and pulp tissue.^7^, ^8^, ^9^ SCAPs are located within the apical papilla tissue, and their migration and establishment within the root canal–pulp complex are crucial to achieving regeneration of the dentin-pulp complex.^7^^,^^10^ In order to promote regeneration of dental pulp tissue, SCAPs must migrate from the apical area to the pulp canal, subsequently proliferate, and differentiate into various cell types, for example, fibroblasts, odontoblasts, and endothelial cells, for dentin-pulp complex regenerations.

According to the aforementioned information, simvastatin promotes odontogenic differentiation, angiogenesis, and dental pulplike tissue formation. Thus, this would be a candidate small molecule to facilitate regenerative endodontic treatment. The investigation regarding the roles of simvastatin on main stem cells in regenerative endodontics (SCAPs) is currently lacking. The present study aims to evaluate the effects of simvastatin on the viability, proliferation, and migration of SCAPs in an *in vitro* setting.

## Materials and methods

### Cell isolation and culture

Immature third molars were obtained from patients scheduled for tooth extraction at the Faculty of Dentistry, Chulalongkorn University, according to their normal treatment plan, with written informed consent obtained. The study protocol was approved by the Human Research Ethical Committee, Faculty of Dentistry, Chulalongkorn University (approval No. 062/2022). After tooth removal, the tissues of the apical papilla located at the apex of the root were collected, and cell explantation was used to isolate cells. The isolated cells were cultured in Dulbecco-modified Eagle medium (DMEM, Gibco) supplemented with 10% fetal bovine serum (FBS, Gibco), 2 mM L-glutamine (GlutaMAX^TM^, Gibco), 100 μg/mL streptomycin, and 100 U/mL penicillin (Sigma-Aldrich) at 37 °C in a humidified atmosphere with 5% CO_2_. To assess stem cell characteristics, an analysis of the expression of cell surface markers and differentiation ability towards osteogenic and adipogenic lineages was performed following established protocols.^11^^,^^12^ For simvastatin treatment, the simvastatin concentration (cat. No. 000008154, Sigma-Aldrich) concentration was administered, ranging from 100 nM to 1000 nM according to previous literature.^4^^,^^13^

### Cell viability assay

The viability of SCAPs was assessed using the LIVE/DEAD Viability/Cytotoxicity Kit (ThermoFisher). The SCAPs were seeded in 24-well plates at a cell density of 5 × 10^4^ cells per well and treated with 100, 250, 500 and 1000 nM of simvastatin. After 24 and 48 hours, cells were washed twice with a phosphate-buffered solution (PBS). Subsequently, cells were stained with PBS containing 0.5 mmol/L calcein-AM and 0.5 mmol/L ethidium homodimer-1 in a light-protected container and incubated for 30 minutes. Excess dye was removed by washing with PBS. The cells were then observed under a fluorescent microscope at 517 nm and 617 nm.

### Cell proliferation assay

The proliferation was examined using the 3-(4,5-dimethylthiazol-2-yl)-2,5-diphenyl-2H-tetrazolium bromide (MTT) (Tocris Bioscience) assay. SCAPs were seeded in 24-well plates at a cell density of 5 × 10^4^ cells per well. Cells were incubated with 0.5 mL/mg of MTT solution for 30 minutes on days 1, 3, and 7 after simvastatin treatment. Subsequently, the formazan crystals were dissolved using a DMSO/glycine buffer. The absorbance of the solubilised dye was measured at 570 nm using a microplate reader.

### Cell apoptosis assay

Cells were seeded in 6-well plates at a cell density of 3 × 10^5^ cells per well and maintained in a growth medium. The cells were stained with an apoptosis detection kit, propidium iodide/annexin V staining (Roche Diagnostics), following the manufacturer's protocol. Subsequently, stained cells were analysed using a FACSCalibur flow cytometer (BD Biosciences Pharmingen).

### Cell cycle analysis

The 3 × 10^5^ cells were seeded in 6-well plates and maintained in a growth medium. Cells were fixed with cold 70% ethanol for 15 minutes, followed by rinsing with PBS and treatment with RNase at room temperature for 30 minutes. Subsequently, cells were stained with PI solution (Sigma-Aldrich) in the dark at room temperature for 30 minutes. Finally, stained cells were analysed using a FACSCalibur flow cytometer (BD Bioscience).

### Cell migration assay

The cells (3 × 10^5^ cells) were seeded in 6-well plates and maintained in a normal growth medium. A sterile pipette tip was scratched on the monolayer of cells to create the *in vitro* wound area. Subsequently, the cells were washed twice with PBS and treated with simvastatin. Images were captured at the marked position at 24 and 48 hours. Image analysis was performed using ImageJ software, and the percentage of area without cells was calculated.

### Colony forming unit assay

A single-cell suspension of 500 cells was seeded in 6-well plates and further cultured in a normal growth medium. On day 12, cells were then fixed in 10% buffered formalin, followed by rinsing twice with PBS. Subsequently, cells were stained with Coomassie Blue. The colonies were then captured under a microscope, and the amount of staining was quantified by destaining with 50% (v/v) methanol in water with 10% (v/v) acetic acid.

### Statistical analysis

Biologic replications were carried out in all experiments with cells from at least 4 donors. Data were presented as mean ± standard error of the mean. The statistical analyses were conducted using Prism8 software (GraphPad Software). The statistical comparison was analysed using the Kruskal–Wallis test, followed by a pairwise comparison test. A *P* value <.05 was considered a statistically significant difference.

## Results

### Characterisation of SCAPs

**Fig. 1:**
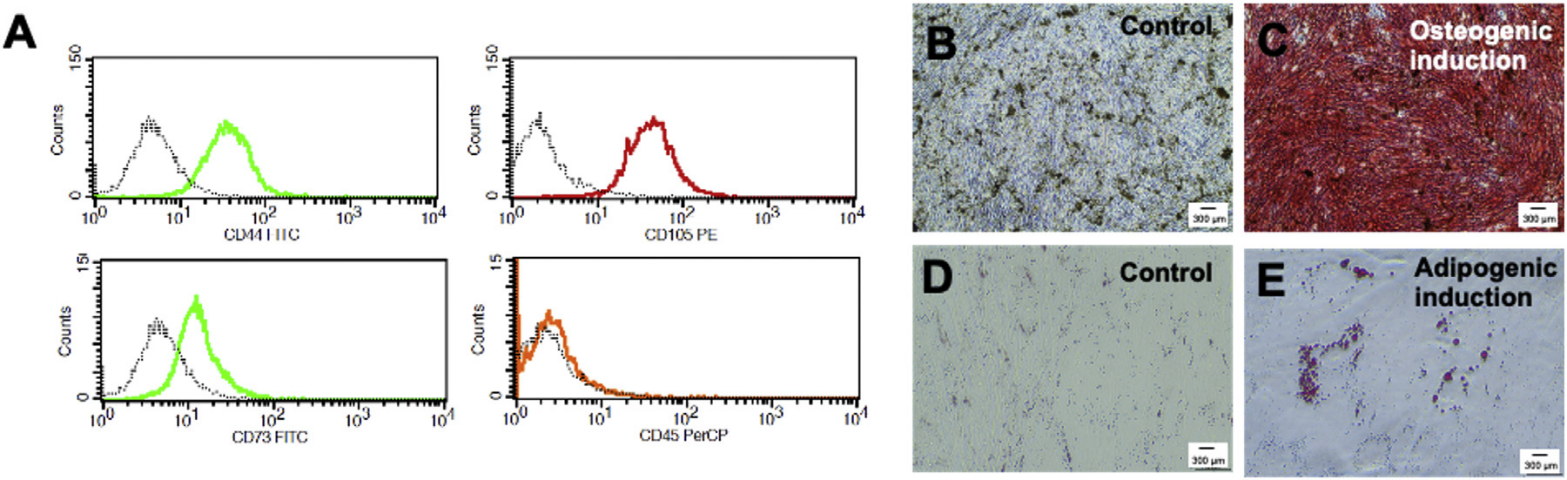
Characterisation of stem cells isolated from apical papilla. **A**, Flow cytometry analysis of stem cell surface markers CD44, CD105, CD73, and CD45. **B** and **C**, Mineral deposition after osteogenic induction for 14 days was examined using Alizarin Red S staining. **D** and **E**, Intracellular lipid accumulation after adipogenic induction for 28 days was determined by Oil Red O staining.

Isolated cells from apical papillae tissue exhibited spindle-shaped morphology and expressed mesenchymal stem cell markers, including CD44, CD73, and CD105, whilst the expression of CD45 was lacking (Figure 1A). In order to prove mesenchymal stem cell characteristics, the differentiation towards osteogenic and adipogenic lineages were investigated. After 14 days of culture in an osteogenic medium, SCAPs demonstrated a substantial increase in mineral deposition, as confirmed by Alizarin Red S staining. The cells cultured in a normal growth medium were used as the control (Figure 1B and C). Further, the intracellular lipid droplet accumulation was markedly noted in those cells maintained in an adipogenic induction medium for 28 days compared with those cells cultured in a normal growth medium (Figure 1D and E). These data confirmed the mesenchymal stem cell characteristics of the isolated cells.

### Simvastatin attenuated cell proliferation and colony formation

**Fig. 2:**
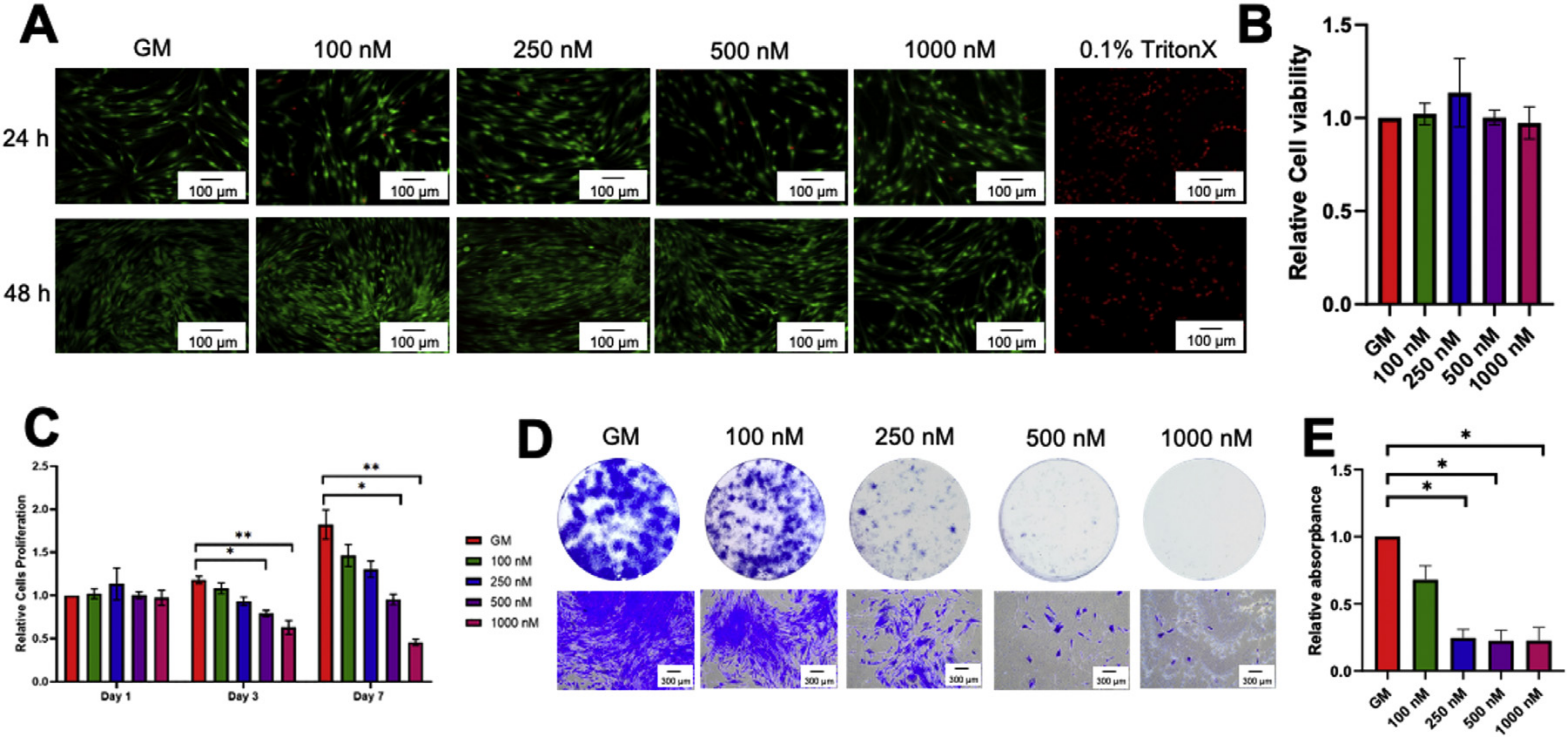
Simvastatin attenuated cell proliferation and colony formation. Stem cells isolated from apical papillae (SCAPs) were exposed to 100, 250, 500, and 1000 nM simvastatin. **A**, Cell viability was assessed using the live/dead assay at 24 and 48 hours. Cells cultured in normal growth medium and 0.1% TritonX were used as negative and positive controls, respectively. **B** and **C**, Cell viability was examined using the 3-(4,5-dimethylthiazol-2-yl)-2,5-diphenyl-2H-tetrazolium bromide (MTT) assay at 24 hours, 3 days, and 7 days. **D**, The cells were seeded in low density and cultured in a normal growth medium for 14 days. The colony formation was examined using Coomassie Blue staining. **E**, The elution of Coomassie Blue dye was measured at absorbance and normalised to the control. The bars indicate a statistically significant difference (*P* < .05). GM, normal growth medium.

SCAPs were treated with simvastatin at concentrations of 100, 250, 500, and 1000 nM in normal growth medium. Cell viability was examined using the live/dead assay at 24 and 48 hours. The positive control for cell death was the condition treated with 0.1% TritonX (Figure 2A). No significant difference in dead cells (red fluorescence–stained cells) was observed comparing all treated conditions with the control. However, cells treated with TritonX exhibited a dramatic increase in cell death. Correspondingly, cell viability was also assessed using an MTT assay at 24 hours (Figure 2B). There was no significant difference in cell viability in all treated conditions compared with the control. These results indicated that all test concentrations of simvastatin did not exhibit immediate cytotoxicity on SCAPs.

Cell proliferation was examined using MTT and colony-forming unit assay. Results demonstrated that simvastatin reduced cell number at later time points in a dose-dependent manner. days 3 and 7, simvastatin at a concentration of 500 and 1000 nM significantly reduced cell number compared with the control group (Figure 2C). Cells were seeded at a low density so as to detect the colony formation ability originated from a single cell. At day 12, simvastatin dramatically reduced the colony number and the cell density in the colony in a dose-dependent manner (Figure 2D). A significant decrease was observed at every treated concentration except for 100 nM compared with the control (Figure 2E).

### Simvastatin induced apoptosis but did not affect cell cycle progression in SCAPs

**Fig. 3:**
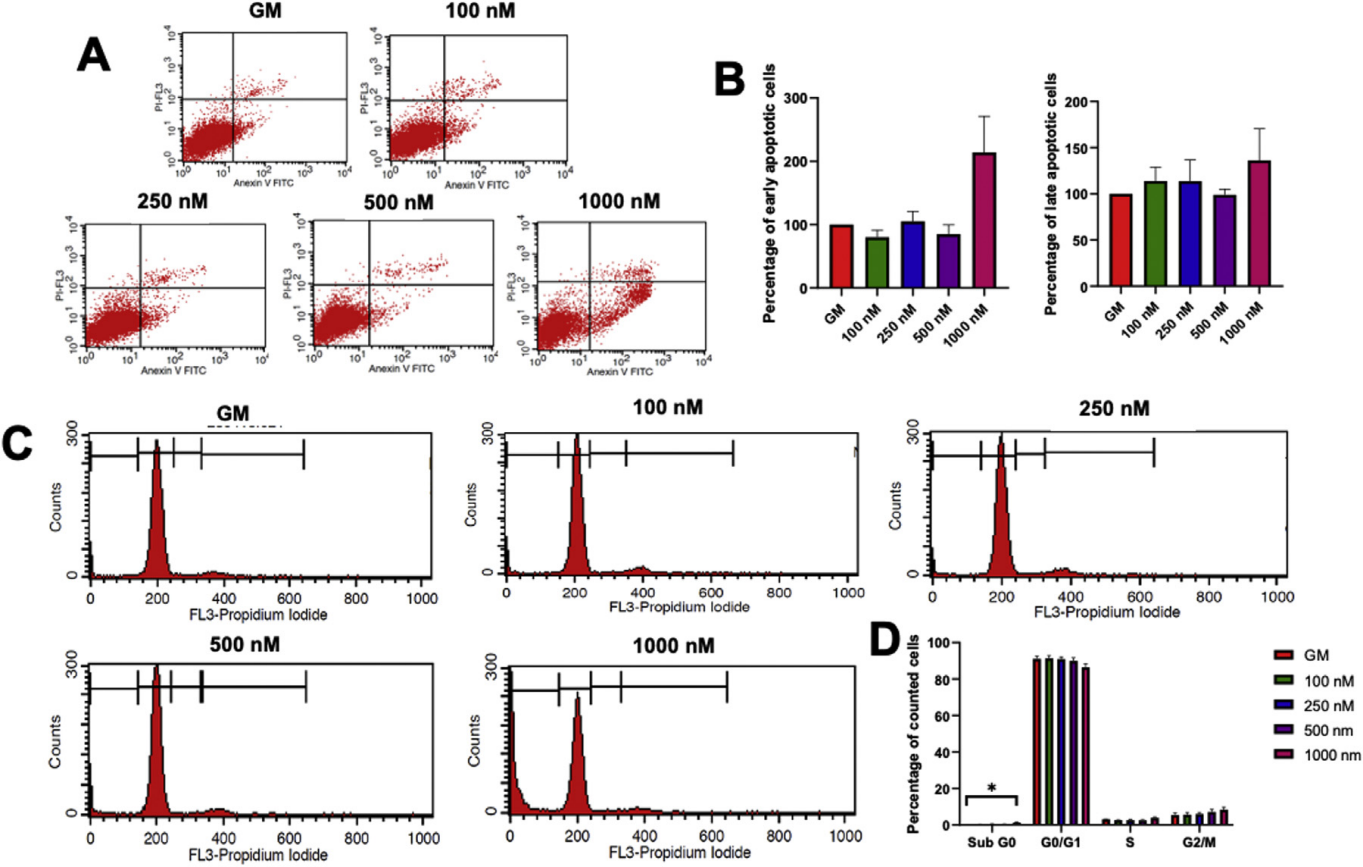
Simvastatin induced apoptosis and affect cell cycle progression. Cells were treated with 100 nM, 250 nM, 500 nM, and 1000 nM simvastatin. **A**, At 7 days, cells were stained with propidium iodide/Annexin V and further analysis was performed using flow cytometry. Representative cytograms of the apoptosis assay were illustrated. **B**, The percentage of early and late apoptotic cells was calculated. **C**, Cell cycle progression was examined using PI staining followed by flow cytometry analysis. Representative histograms were shown to demonstrate the cell count in each step of the cell cycle on day 7. **D**, The percentage of cell cycle subpopulations was demonstrated. The bars indicate a statistically significant difference (*P* < .05). GM, normal growth medium.

Simvastatin at 1000 nM induced noticeable early-stage cell apoptosis in SCAPs at day 7 (Figure 3A and B). The PI staining for cell cycle assay revealed a significant increase in the subG0 population with a slight decrease in the G0/G1 population in the 1000 nM simvastatin-treated condition (Figure 3C and D).

### Simvastatin reduced SCAP migration

**Fig. 4:**
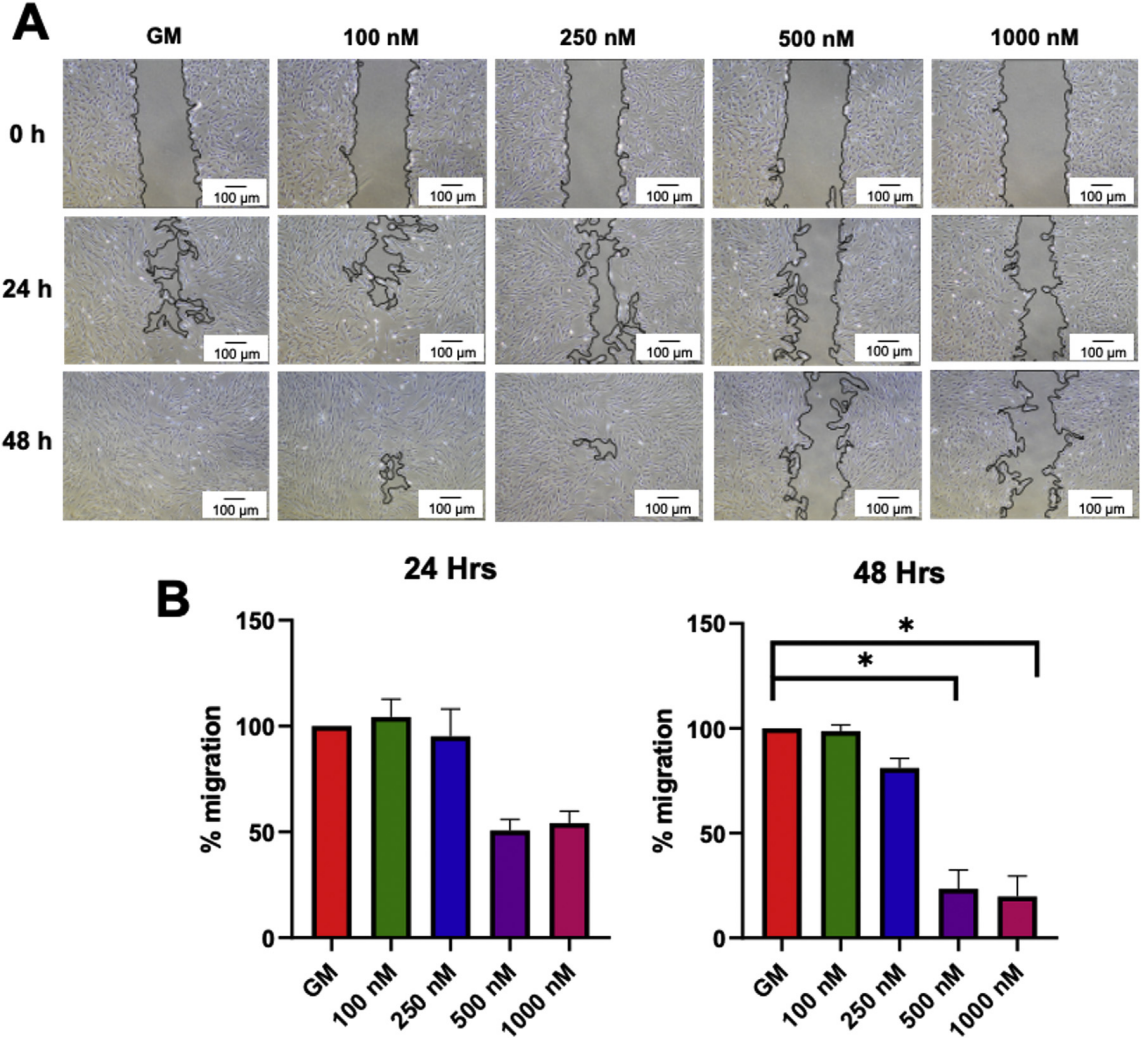
Simvastatin reduced the migration of stem cells isolated from apical papillae (SCAPs). Cells were treated with 100 nM, 250 nM, 500 nM, and 1000 nM simvastatin. **A**, Representative images of cell migration were illustrated at 0, 24, and 48 hours. **B**, Graphs demonstrated the percentage of cell migration calculated from the wound closure area at 24 and 48 hours. The bars indicate a statistically significant difference (*P* < .05). GM, normal growth medium.

*In vitro* scratch assay. At 24 hours, the groups treated with 500 nM and 1000 nM of simvastatin showed a notable decreased cell migration rate compared to the control group, although no statistically significant difference was observed. Furthermore, the concentration of 500 nM and 1000 nM significantly inhibited cell migration rate at 48 hours (Figure 4A and B).

## Discussion

The present study centres on the effects of simvastatin on SCAPs, exploring their viability, proliferation, apoptosis, cell cycle, and migration. Whilst simvastatin has been previously studied in various contexts, its precise impact on SCAPs, particularly in the context of regenerative endodontics, has not been comprehensively addressed. Our research fills a critical knowledge gap by providing detailed insights into how simvastatin influences these essential cellular processes, which are crucial for dental pulp tissue regeneration.

The key findings of this study demonstrate that high concentrations of simvastatin have detrimental effects on the proliferation, viability, and migration capacity of SCAPs. In the context of regenerative endodontics, it is crucial that SCAPs migrate from the apical papilla tissue into the root canal–pulp complex, initiated by active bleeding induction, and proliferate to initiate the regeneration process. Therefore, the use of simvastatin as an adjunctive medication in regenerative endodontics based on our study's results may not be ideal, as it could potentially interfere with the migration and proliferation of SCAPs, which are essential for the regeneration process.

The present study reported that simvastatin inhibited cell proliferation and colony-forming unit ability in a dose-dependent manner ranging from 100 to 1000 nM. Significant reduction in cell number at a later time point was markedly observed at a high concentration (500 and 1000 nM). However, the effect of simvastatin on colony formation was observed at 250 nM. In a previous report on mesenchymal stem cells, simvastatin reduced the number of mesenchymal stem cells in a dose-dependent manner ranging from 0.05 to 5 μM.^14^ Further, 0.01 to 0.1 μM simvastatin significantly promoted—but 1 μM simvastatin inhibited—colony formation and cell proliferation in nucleus pulposus–derived mesenchymal stem cells.^13^ Dramatic decreases in cell numbers were observed as early as 4 days after simvastatin treatment.^14^ In DPSCs, simvastatin 1 μM also inhibited cell proliferation in a dose-dependent manner.^4^ However, a significant decrease in cell number was observed in those cells treated with 10 μM simvastatin. On the contrary, simvastatin at 1 μM promoted cell proliferation in human dental pulp stem cells, which occurred through the PI3K/AKT pathway.^15^ In human periodontal ligament (PDL) cells, simvastatin dose-dependently increased cell metabolism, showing significant effects at 0.1 μM and 1 μM concentrations after 24 hours, except for a reduction at 1 uM after 72 hours, according to the MTT assay. Additionally, the BrdU assay also shows that simvastatin notably promoted human PDL cell proliferation at concentrations of 10 nM, 100 nM, and 1000 nM after 24 hours, with peak effects at 100 nM and 1000 nM. No significant difference was observed at 10 μM. After 72 hours, significant differences were generally absent, except for a decrease observed at the highest concentration.^16^

High concentrations of simvastatin at 1000 nM promoted apoptosis on day 7 after simvastatin treatment. There was no marked change in cell cycle progression. A study in mesenchymal stem cells revealed that 5 μM simvastatin treatment increased annexin V stained cells on day 18, corresponding to the change in cell morphology, which implies the induction of cell apoptosis.^14^ The effects of different doses on cell apoptosis were investigated in mesenchymal stem cells derived from the nucleus pulposus. The results demonstrated that 0.01 to 0.1 μM simvastatin significantly attenuated but 1 μM simvastatin induced cell apoptosis in nucleus pulposus–derived mesenchymal stem cells.^13^ In the in vitro inflammation condition, simvastatin synergistically increased cell apoptosis in DPSC treated with lipopolysaccharide (LPS).^17^ On the other hand, 1 μM simvastatin did not affect cell apoptosis but caused cell cycle arrest, as determined by the decrease in the population of G2/M4.

Simvastatin attenuated cell migration in the in vitro scratch assay in a dose-dependent manner in SCAPs. The role of simvastatin in the attenuation of cell migration and invasion is in cancer cells. Simvastatin at a concentration of 0.01 to 10 μM inhibited gingival fibroblast cell migration in the scratch assay in a dose-dependent manner 16 hours after treatment.^18^ This may benefit the regeneration process by preventing these cells from occupying space in the root canal that should be reserved for SCAPs. The attenuation of cell migration by simvastatin could be due to the disruption of focal adhesion by altering the cytoskeleton and focal adhesion assembly. In contrast, a study in rat bone marrow mesenchymal stem cells exhibited that simvastatin-loaded mesoporous hydroxyapatite microspheres promoted cell migration in the transwell migration assay in a dose-dependent manner (0.6–60 μg/mL).^19^ Further, simvastatin enhanced SDF-1α–induced migration of bone marrow–derived mesenchymal stem cells in the transwell migration assay.^20^

In contrast to simvastatin's negative results in this study, metformin is a good example of a more ideal adjuvant to be used in regenerative endodontics. Qin et al^21^ investigated the effects of metformin on dental pulp cells (DPCs) and its implications for dentin regeneration. Metformin demonstrated a noncytotoxic nature, with no significant impact on cell proliferation or overall viability of DPCs. Notably, metformin upregulated the expression of critical odontoblastic genes, including *DSPP, DMP1, RUNX2*, and *OCN* mRNA. These findings strongly indicate that metformin has the potential to enhance the differentiation of DPCs into odontoblasts. These results showed the example of favourable properties of metformin for use as an adjuvant in regenerative endodontics.^21^

Simvastatin, when combined with the chitosan-calcium-simvastatin scaffold (CH-Ca-SV scaffold), shows promise in enhancing dentin regeneration. It promotes mineralised tissue formation and supports odontoblastic marker expression, making it a valuable candidate for dental pulp regeneration. The sustained release of simvastatin from the scaffold improves its chemoattractant potential, enhancing its role in dentin regeneration.^22^

The discrepancy regarding the effects of simvastatin on cell responses could be due to the different concentrations of simvastatin used in different studies. Some studies used simvastatin as adjuvant molecules in the combination of treatment regimens, for example, with hydroxyapatite microspheres, methacrylated gelatin microspheres, and SDF-1α. In addition, different cell types could respond differently to stimuli. For example, we note that the effects of simvastatin on cell proliferation and apoptosis in SCAPs differ from those reported in DSPCs. DPSCs and SCAPs exhibit different cellular characteristics and properties.^12^^,^^23^ Therefore, the study in SCAPs provides valuable data on the benefit of simvastatin in those cells that are mainly responsible for regenerative endodontic treatment. However, it should be noted that these findings may differ from the clinical setting, where other factors, such as growth factors released from dentin, can influence the properties and stemness of SCAPs. Furthermore, the complex structure of the apical tissue may influence different cell responses in vivo.

In conclusion, simvastatin still holds potential as an inductive component in the promotion of odontogenic differentiation and further enhances reparative dentin formation by DPSCs. However, the use of simvastatin in regenerative endodontics targeted to modulate the function of SCAPs may not be appropriate, as it exhibits various negative effects on the proliferation, viability, and migration of SCAPs. However, it can be used as a pulp capping material and alveolar bone regeneration since some studies show a positive effect on DPSCs and mesenchymal stem cells and that it can induce cell proliferation and migration and promote osteogenic differentiation in these cells. Further investigation into the osteo-/odontogenic inductive potential of simvastatin in SCAPs and the appropriate order of combination of irrigants is necessary to confirm its usefulness in regenerative endodontics.

## Conflict of interest

None disclosed.
